# EFFECT OF EMOTION NAMING ON EMOTION REGULATION IN YOUNGER AND OLDER ADULTS

**DOI:** 10.1093/geroni/igad104.0112

**Published:** 2023-12-21

**Authors:** Hannah Wolfe, Derek Isaacowitz

**Affiliations:** Northeastern University, Boston, Massachusetts, United States; Northeastern University, Boston, Massachusetts, United States

## Abstract

Prior research has shown that naming one’s emotions before regulation has a detrimental effect on regulation success (Nook et al., 2020). These researchers argued this effect was due to the affect crystallization that occurs after naming an emotion, which in turn makes it harder to modify or regulate that emotion. The aim of the current replication-extension study was to evaluate whether this effect would replicate in an older adult sample. Due to age-related changes in emotion regulation knowledge and behaviors, we hypothesized that age would moderate this effect, such that older adults would not be detrimentally impacted by naming their emotions, but younger adults would. Younger (18-25 years, N=50) and older adults (60+ years, N=50) viewed 80 IAPS images while being told to first ‘look’ or ‘name’ their experienced emotion and second to ‘look’ or ‘regulate’ their emotions (creating four within-subjects conditions). Unpleasant affect was measured on each trial, analyzed via a 2(age group) X 2(naming: yes/no) X 2(regulating: yes/no) mixed ANOVA. Results suggest a significant three-way interaction; within both age groups, the name-regulate trials were associated with significantly greater unpleasant affect compared to the look-regulate trials, suggesting that naming emotions was detrimental for both age groups. However, contrary to our hypothesis, this effect was larger in older adults (d=.82) than younger adults (d=.41). These findings suggest that affect crystallization may be stronger or faster in older adulthood.

